# Fusing an exonuclease with Cas9 enhances homologous recombination in *Pichia pastoris*

**DOI:** 10.1186/s12934-022-01908-z

**Published:** 2022-09-07

**Authors:** Kun Zhang, Xingpeng Duan, Peng Cai, Linhui Gao, Xiaoyan Wu, Lun Yao, Yongjin J. Zhou

**Affiliations:** 1 Division of Biotechnology, Dalian Institute of Chemical Physics, Chinese Academy of Sciences, Dalian, 116023 China; 2 Henan Engineering Laboratory for Bioconversion Technology of Functional Microbes, College of Life Sciences, Henan Normal University, Xinxiang, 453007 Henan China; 3 College of Life Sciences, Liaoning Normal University, Dalian, 116029 Liaoning China; 4 University of Chinese Academy of Sciences, Beijing, 100049 China; 5 Dalian Key Laboratory of Energy Biotechnology, Dalian Institute of Chemical Physics, Chinese Academy of Sciences, Dalian, 116023 China; 6 CAS Key Laboratory of Separation Science for Analytical Chemistry, Dalian Institute of Chemical Physics, Chinese Academy of Sciences, Dalian, 116023 China

**Keywords:** *Pichia pastoris*, CRISPR/Cas9, Gene editing, Homology-directed repair, Metabolic engineering

## Abstract

**Background:**

The methylotrophic yeast *Pichia pastoris* is considered as an ideal host for the production of recombinant proteins and chemicals. However, low homologous recombination (HR) efficiency hinders its precise and extensive genetic manipulation. To enhance the homology-directed repair over non-homologous end joining (NHEJ), we expressed five exonucleases that were fused with the Cas9 for enhancing end resection of double strand breaks (DSBs) of DNA cuts.

**Results:**

The endogenous exonuclease Mre11 and Exo1 showed the highest positive rates in seamless deletion of *FAA1*, and fusing the *MRE11* to the C-terminal of *CAS9* had the highest positive rate and relatively high number of clones. We observed that expression of *CAS9-MRE11* significantly improved positive rates when simultaneously seamless deletion of double genes (from 76.7 to 86.7%) and three genes (from 10.8 to 16.7%) when overexpressing *RAD52*. Furthermore, *MRE11* overexpression significantly improved the genomic integration of multi-fragments with higher positive rate and clone number.

**Conclusions:**

Fusion expression of the endogenous exonuclease Mre11 with Cas9 enhances homologous recombination efficiency in *P*. *pastoris*. The strategy described here should facilitate the metabolic engineering of *P*. *pastoris* toward high-level production of value-added compounds.

**Supplementary Information:**

The online version contains supplementary material available at 10.1186/s12934-022-01908-z.

## Background

**Fig. 1:**
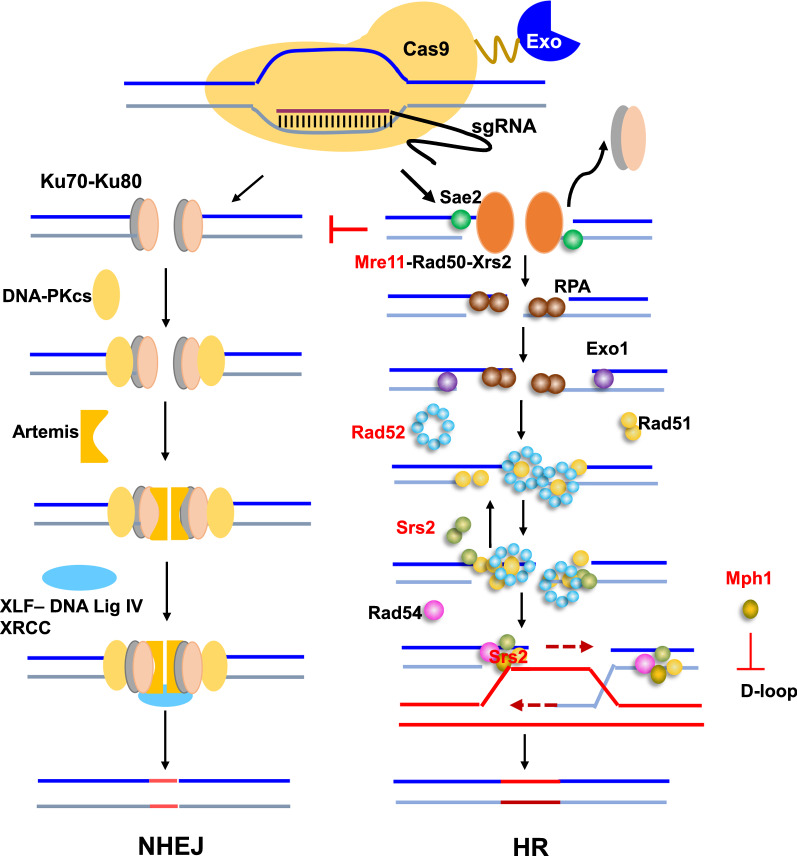
Homologous recombination process mediated by CRISPR/Cas9. The fusion of an exonuclease and Cas9 is advantageous in promoting the exonuclease to bind with DSBs, which subsequently initiates resection of the DSBs toward HR and inhibits NHEJ pathway. The genes marked as red were overexpressed or deleted for enhancing HR efficiency

*Pichia pastoris* (*Komagataella phaffii*) is an excellent chassis cell used for protein production and chemicals synthesis [1–4]. In addition, *P*. *pastoris* has high potential for sustainable bio-manufacturing with utilizing methanol as a substrate, which should be helpful for carbon neutrality [5]. Construction of efficient cell factory requires extensive metabolic rewiring with consequent multiple genes manipulation [6–9]. Currently, the CRISPR/Cas9 system has been successfully applied for genome editing in *P*. *pastoris* and other organisms [10–14]. However, the dominant NHEJ over HR in *P*. *pastoris,* during the repairing DSBs that generated by Cas9 cutting, brings the challenges in precise genetic manipulation such as seamless gene deletion and targeted gene integration (Fig. 1). Recently, we and others tried to engineer the recombination machinery to improve the HR repair process by overexpressing the HR related genes such as *RAD52* and/or knocking out the NHEJ genes *KU70/80* [11, 12, 15–17]. However, the low efficiency in simultaneous deletion of multiple genes requires further enhancing HR procedure.

The choice of DSBs repair pathway between NHEJ and HR mainly depends on the initial processing of the DSBs ends [18, 19]. Generally, initial resection of the HR is trigged by Sae2 protein and the Mre11-Rad50-Xrs2 (MRX) complex in yeast. DSBs end resection is further extended by long-distance exonucleases Exo1 and Dna2 [20]. MRX and other factors work together to generate short 3′ overhangs, which is necessary for initiating HR. Additionally, we previously identified that the nuclease ScSae2 can promote the HR efficiency of *GUT1* [17]. We thus explored to initiate the exonuclease mediated DNA end resection for enhancing the HR and repressing the NHEJ, and fusing an exonuclease with Cas9 might immediately initiate the formation of 3′ overhangs upon DSBs generation by Cas9 cutting (Fig. 1).

Here, we tried to fuse various exonucleases to Cas9 to enhance the HR process in *P. pastoris* by accelerating the formation of 3′ overhangs following DSBs generation. With this genetic engineering platform, we improved the simultaneous deletion of multiple genes and integration of multiple fragments, which should be useful for metabolic engineering of *P. pastoris* and even other non-conventional yeasts.

## Results

### Enhancing HR efficiency by expressing a fusion of exonuclease and Cas9

**Fig. 2:**
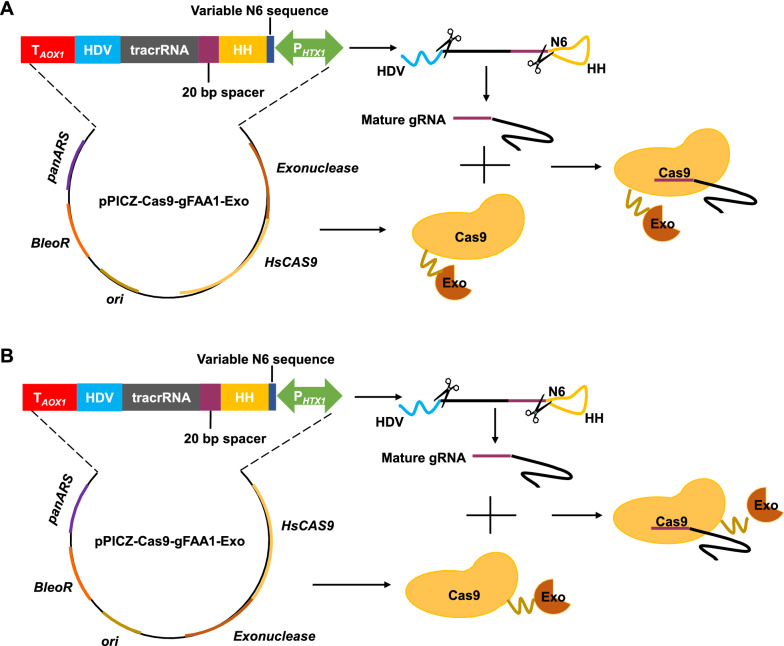
Schematic representation of the fusion expression of Cas9 and exonucleases. A bidirectional promoter P_*HTX1*_ was used to express the *CAS9* gene, exonuclease genes and the gRNA. The gRNA was flanked by hammerhead (HH) and hepatitis delta virus (HDV) ribozymes. These two ribozymes were transcribed and auto-catalytically cleaved themselves to generate a mature gRNA. Exonucleases were fused to N-terminus (**A**) or C-terminus (**B**) of Cas9 with a linker (GGGGS)_3_, respectively

Exonucleases can cleave DSBs to form single-stranded DNA, we thus tried to fuse exonuclease with Cas9 to initiate the HR based repair immediately upon Cas9 cutting. To select the suitable exonuclease, we fused five exonuclease-encoded genes, phage T7 exonuclease (*T7Exo*), phage λ Red recombination system exonuclease (*λRedExo*), *Escherichia coli* exonuclease III (*EcExo III*), and endogenous *MRE11* and *EXO1* to the N-terminal or C-terminal of *CAS9* on the plasmids, respectively (Fig. 2). We compared the HR efficiency by quantifying the positive rate of seamless deletion of *FAA1* (encoding fatty acyl-CoA synthase 1), since *FAA1* deletion efficiency was low in wild-type of *P*. *pastoris* GS115 [17].

**Fig. 3:**
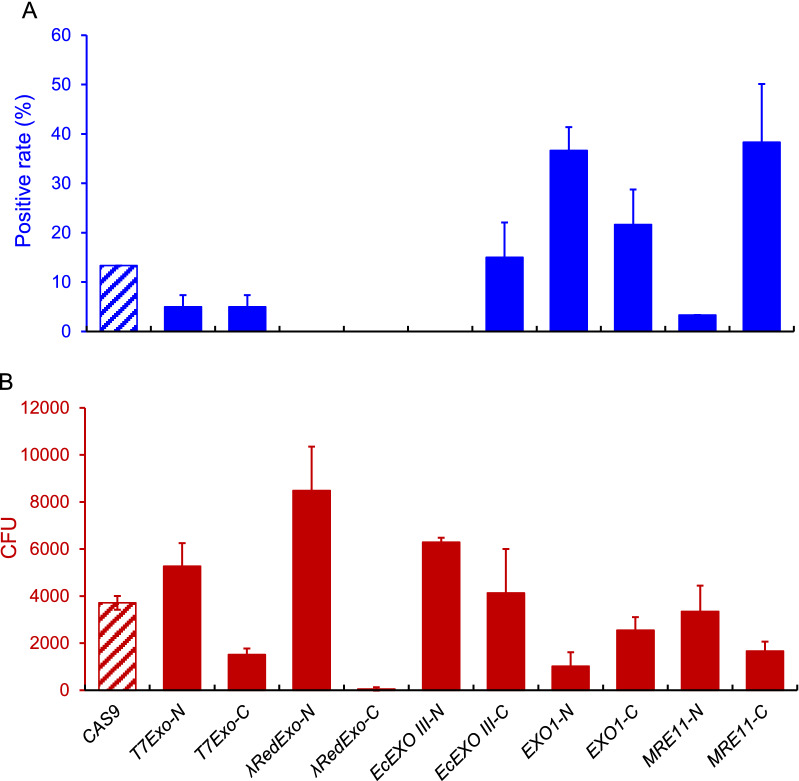
Screening exonucleases to improve HR efficiency. The positive rate (**A**) and CFU (**B**) for fusing five exonucleases to the N or C-terminus of Cas9. Exonucleases were employed to enhance HR efficiency at the *FAA1* locus in the *P*. *pastoris* GS115 strain. Sixty clones from two plates were randomly picked for PCR analysis. The positive rate was from the number of correct clones divided by the total number of picked clones. The CFU numbers were calculated from the total colonies on the plates. Data are presented as means of two biologically independent samples

The endogenous *MRE11* and *EXOI* significantly improved the positive rates by 25% and 23.4%, respectively, while the control with only expressing *CAS9* had a positive rate of 13.3%. In particular, fusing expression of *MRE11* to the C-terminal of *CAS9* (*MRE11*-C) resulted in the highest positive rate of 38.3% (Fig. 3A). It is worthy to mention the position of exonuclease resulted in the big variance of positive rates, which might be attributed to difference in enzyme activity or the structure interface between Cas9 and exonuclease. Considering the highest positive rate and relative sufficient colony forming units (CFU) number, the C-terminal fusion of Mre11 (*MRE11-C*) to Cas9 was used for further genetic manipulation (Fig. 3B).

### Synergistic effect of MRE11 and RAD52 for enhancing HR

**Fig. 4:**
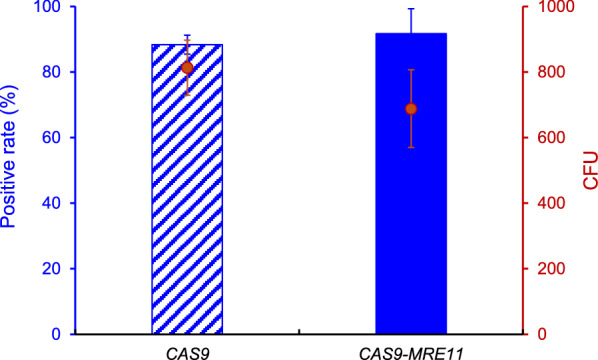
The effect of *MRE11* and *RAD52* overexpression on seamless deletion of *FAA1* gene. 1000 ng DNA donor was used for *FAA1* gene deletion. Twenty clones from each plate were picked for colony PCR analysis. Data are presented as mean ± s.d. with three biologically independent samples

We previously showed that overexpressing endogenous *RAD52* significantly improved HR [17]. We here tried to combine the *MRE11* and *RAD52* for further enhancing HR. Overexpression of *RAD52* had a high positive rate of 88.3% and further fusing the expression of *MRE11* to C terminal of *CAS9* (*CAS9-MRE11*) had a higher positive rate of 91.7% when seamlessly deleting *FAA1*. Consistently, the CFU number of *CAS9-MRE11* decreased slightly (Fig. 4). *RAD52* overexpression significantly improved the manipulation of single genes [17], but precise engineering of multiple genes is still challenging. We thus investigated the synergistic influence of *RAD52* overexpression coupled with the *MRE11* fusion to Cas9 for simultaneously manipulation of multiple genes.

### Simultaneous deletion of multiple genes

**Fig. 5:**
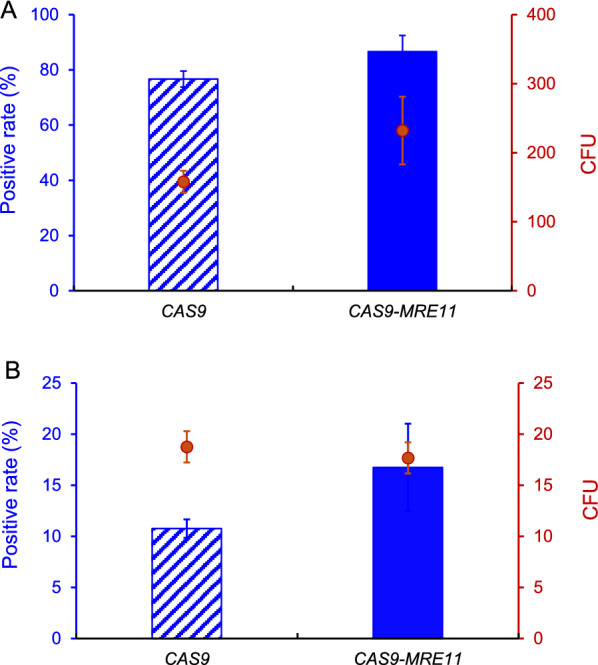
The effect of *MRE11-CAS9* and overexpression of *RAD52* on simultaneous deletion of multiple genes. **A** Simultaneously deleting two genes *FAA2* and *HFD1*. **B** Simultaneously deleting three genes *FAA2, HFD1*, and *POX1*. Deletion of multiple genes was carried out in the *RAD52* overexpression strain GS115-*RAD52*, which was transformed with 500 ng each donor DNA, and plasmids expressing gRNA and *CAS9*/*CAS9-MRE11*. Twenty clones from each plate were picked for colony PCR analysis. Data are presented as mean ± s.d. with three biologically independent samples

We firstly applied our system for simultaneous deletion of multiple genes, which should be helpful for extensive metabolic engineering. Fusion expression of *CAS9-MRE11* resulted in a high positive rate of 86.7% when simultaneously deleting *FAA2* and *HFD1*, which was higher than that of the control (*CAS9*, 76.7%) in the *RAD52* overexpression strain GS115-*RAD52*. Interestingly, *CAS9-MRE11* significantly increased the CFU number (Fig. 5A). In simultaneous deletion of three genes (*FAA2*, *HFD1* and *POX1*), *CAS9-MRE11* also improved the positive rates (from 10.8% of the control *CAS9* to 16.7%) with the similar CFU numbers (Fig. 5B). These results suggested that fusing Mre11 with Cas9 helped to initiate the HR based repair of multiple DSBs and thus improved the seamless deletion of multiple genes.

### CAS9-MRE11 enhances genomic integration of multi-fragments

**Fig. 6:**
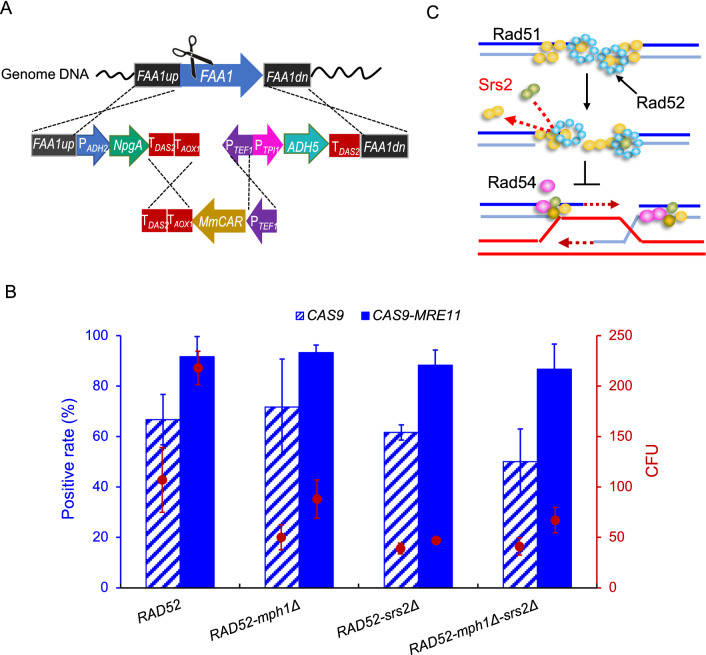
The effect of *CAS9-MRE11* on the integration of multi-fragments. **A** The fatty alcohol biosynthetic pathway was integrated into *FAA1* locus. This pathway was divided to the three parts with overlapping 500 bp at the promoter or terminator regions and 1000 bp at homologous arms. **B** Integration efficiency of *CAS9-MRE11* on multi-fragment in the background strains *RAD52*, *RAD52-mph1Δ*, *RAD52-srs2Δ*, and *RAD52-mph1Δ-srs2Δ*. 500 ng of each donor DNA was used for evaluating integration efficiency. Twenty clones from each plate were picked for colony PCR analysis. Data are presented as mean ± s.d. with three biologically independent samples. **C** Schematic diagram of Srs2 protein inhibiting HR process by interfering with Rad51

Multi-fragments assembly is useful for integrating long biosynthetic pathways. We previously observed that overexpressing *RAD52* and deleting *MPH1* contributed to the integration of three DNA fragments into specific genomic locus of *P. pastoris* [17]*.* We here decided to further investigate if *CAS9-MRE11* expression could enhance the integration of multi-fragments (total 11 kb) of a fatty alcohol biosynthetic pathway (Fig. 6A). In the strain GS115-*RAD52*, *CAS9-MRE11* improved the positive rate to 91.7%, in comparison to the control of 66.7% (Fig. 6B). In the background strain GS115-*RAD52-mph1Δ*, *CAS9-MRE11* also resulted in a significant higher positive rate of 93.3% compared with the control (71.7%). Remarkably, *MRE11* overexpression increased CFU numbers by 103.7% and 76% in these two genetic backgrounds.

The Yeast Srs2 protein is a 3′–5′ DNA helicase and negative regulator of Rad51 function. It removes Rad51 filament and thereby inhibit HR (Fig. 6C) [21, 22]. We thus tried to delete *SRS2* for enhancing HR, which however resulted in lower positive rates during the integration of multi-fragments. But expressing *CAS9-MRE11* still improved the positive rates and CFU numbers in the background strains GS115-*RAD52*-*srs2Δ* and GS115-*RAD52-mph1Δ*-*srs2Δ* (Fig. 6B).

## Discussion

We here found that fusing an endogenous exonuclease Mre11 with Cas9 significantly improved the HR process in *P. pastoris*, which facilitated the genetic manipulation including seamless gene deletion, simultaneous deletion of multiple genes and integration of multiple fragments.

As a component of MRX complex, Mre11 possesses both single-stranded DNA endonuclease activity and 3′-to-5′ exonuclease activity. Mre11 is responsible for short-range resection of DSBs with other HR factors like Sae2/CtIP and directing the repair choice towards HR [19, 20]. Fusion of *MRE11* to *CAS9* can accelerate recruitment of the other component of MRX to DSBs, which forces Mre11 to occupy the cleavage site and keeps Ku70/80 dimer away from DSBs (Fig. 1). Thus, this strategy can help to enhance HR process without disrupting NHEJ procedure. Previous studies showed that interfering with the NHEJ pathway, especially deletion of *KU70Δ*, resulted in genetic instability of chromosomes [17], sensitive to UV rays, negative effect on the cell growth and production [23–25]. Furthermore, methanol biotransformation in *P. pastoris* will suffers the methanol toxicity that might bring chromosome damage and the NHEJ pathway can help cells restore their viability rapidly. Thus, the strategy described here will not cause potential adverse effects on cellular fitness and will be helpful for constructing robust cell factory.

We and others previously showed that overexpression of HR machinery genes such as *RAD52* remarkably enhance the HR efficiency in yeast and mammalian cells [15, 17, 26, 27], which however resulted in low colony numbers and brought the difficulties in picking out the positive clones. One possible reason is that HR is considerably slower than NHEJ and the NHEJ can save more cells from DSB event. NHEJ requires little or no end resection to directly reconnect the DSB ends. In contrast, HR requires short resection and long resection of DSBs and the donor to implement the repair process. In addition, other proteins are also likely to be limiting factors in the HR repair pathway [19, 20]. We here found that fusing *MRE11* with *CAS9* improved the CFU numbers during simultaneous deletion of two genes and integration of multi-fragments (Figs. 5A and 6), suggesting that Mre11 is conducive to initiate the HR based repair of multiple DSBs rapidly. Improving CFU numbers should be helpful for picking out the positive clones during multiplex gene editing and pathway construction. These results also suggested that *MRE11* and *RAD52* had a synergy in enhancing HR in *P. pastoris*.

D-loops are vital intermediates of HR. Most nascent D-loops are impaired by two pathways: one mediated by the Srs2 helicase and the other involved in the Mph1 helicase and the Sgs1-Top3-Rmi1 complex [28]. *SRS2* deletion does not give rise to increase of HR efficiency, which might be attributed to the complex regulation of cell multi-invasion-induced rearrangement process [29]. Finally, we anticipated that elucidating an explicit role of protein Srs2 will favor regulation of HR in further applications.

In conclusion, we showed that fusing the endogenous *MRE11* with *CAS9* can increase HR efficiency in *P. pastoris,* which enhanced precise manipulation of multiple genes and might be applied to other non-conventional microbes.

## Methods and materials

### Strains, media, and cultivation conditions

*P*. *pastoris* GS115 and its derived strains were cultivated in YPD medium (20 g/L glucose, 10 g/L yeast extract and 20 g/L peptone) at 30 ℃. For screening of transformants, YPD medium was supplemented with 100 mg/L zeocin. *E*. *coli* DH5α was grown in LB medium (10 g/L tryptone, 10 g/L NaCl, and 5 g/L yeast extract) at 37 ℃ and used for plasmids construction. 25 mg/L zeocin was added to select and maintain plasmids. All strains used in this study are listed in Table 1.

**Table 1:** Strains used in this study

Strain	Genotype	Reference
DH5α	F^−^, φ80d*lac*ZΔM15, Δ(*lacZYA-argF*)U169, *deoR*, *recA1*, *endA1*, *hsdR17(*$r_{k^{-}}$*, *$m_{k^{+}}$*)*, *phoA*, *supE44*, *λ*^*−*^, *thi-1*, *gyrA96*,* relA1*	Takara
GS115	*Mut*^+^, *his4*^*−*^, *AOX1*, *AOX2*	Lab stock
GS115-*RAD52*	GS115, *HIS4*::P_*GAP*_ -*PpRAD52*-T_*AOX1*_	[17]
GS115-*RAD52*-*mph1Δ*	GS115, *HIS4*::P_*GAP*_ -*PpRAD52*-T_*AOX1*_, *mph1Δ*	[17]
GS115-*RAD52*-*srs2Δ*	GS115, *HIS4*::P_*GAP*_ -*PpRAD52*-T_*AOX1*_, *srs2Δ*	This study
GS115-*RAD52*-*mph1Δ-srs2Δ*	GS115, *HIS4*::P_*GAP*_ -*PpRAD52*-T_*AOX1*_, *mph1Δ*, *srs2Δ*	This study

### Plasmids construction

All plasmids and primers used in this study were listed in the Additional file 1: Table S1 and S2. Plasmid construction was performed as previously described [17]. Most gRNA expression plasmids were constructed based on pPICZ-Cas9-gFAA1, which includes a *CAS9* gene and HH-*FAA1*-gRNA-HDV. The genes encoding phage T7 exonuclease (*T7Exo*), phage λ Red system exonuclease (*λRedExo*) *E. coli* exonuclease III (*EcExo III*) were codon optimized and synthesized by GENEWIZ (Suzhou, China) (Additional file 1: Table S3). Two endogenous genes *MRE11* and *EXO1* were obtained by PCR. These exonuclease genes were fused to the N or C-terminus of *CAS9* gene with a linker (GGGGS)_3_. For gRNA expression plasmids, three fragments including vector backbone, *CAS9* and the exonuclease genes were ligated with ClonExpress II One Step Cloning Kit (Vazyme Biotech Co., Ltd, Nanjing, China). The plasmid pPICZ-Cas9-gSRS2 was constructed with primers SRS2-sgRNA-F/Backbone-DR and SV40-DF/SRS2-sgRNA-R.

### Seamless gene deletion

*FAA1*, *FAA2*, *HFD1*, *POX1* and *SRS2* gene was seamlessly deleted by the corresponding gRNA plasmid and the donor DNA. The upstream and downstream homologous arms (HAs) with a length of 1000 bp were amplified from genome, and then were fused by overlap extension PCR to form a donor DNA for seamless deletion.

DNA transformation was carried out with a condensed electroporation protocol [30]. Briefly, approximately 4 μL DNA (500 ng plasmid and 500–1000 ng donor DNA) and 40 μL of competent cells were mixed in an electroporation cuvette and then incubated for 2 min on ice. Samples were electroporated with Gene Pulser® II electroporator (Bio-Rad Laboratories, Hercules, CA, USA) using the following parameters: 2.0 mm cuvette gap, 1500 V charging voltage, 200 Ω resistance, 25 μF capacitance. Immediately after electroporation, samples were resuspended in 0.5 mL YPD and 0.5 mL sorbitol (1.0 M), and incubated in a shaker (30 °C, 220 rpm) for 1 h. The transformed cells were grown for three to four days on YPD plates containing 100 mg/L Zeocin. Total clones on the plates were counted to obtain the CFU number. Twenty clones were randomly picked from each plate for identification of positive clones unless otherwise noted. The deletion and integration manipulations in the genome were confirmed with colony PCR, and the genomic DNA was extracted according to a described protocol [31]. The positive rate was calculated by dividing the number of correct colonies by the total number of picked colonies.

### Multi-fragment integration at the FAA1 gene site

A fatty alcohol biosynthetic pathway of 11 kb was divided into three expression cassettes. *MmCAR*, *npgA* and *ADH5* gene was driven by promoters P_*ADH2*_, P_*TEF1*_ and P_*TPI1*_, respectively. The HAs length at both ends was 1 000 bp, and the other two HAs were about 500 bp (Fig. 5A). These gene expression cassettes were assembled by overlap extension PCR, and simultaneously integrated into *FAA1* site using the gRNA plasmid pPICZ-Cas9-gFAA1 or pPICZ-Cas9-PpMre11-gFAA1. 500 ng each double-stranded DNA expression cassette as donors and 500 ng plasmids were transformed to yeast cells.

## Supplementary Information

**Additional file 1: Table S1**. Main plasmids used in the study. **Table S2**. Primers used in this study. **Table S3**. Synthesized gene sequence.

## Abbreviations

DSBs: Double strand breaks
HR: Homologous recombination
NHEJ: Non-homologous end joining
CFU: Colony forming units
MRX: Mre11-Rad50-Xrs2
