# Supplementary material for: Fusing an exonuclease with Cas9 enhances homologous recombination in Pichia pastoris
Source: Microb Cell Fact. 2022 Sep 7;21:182. doi: 10.1186/s12934-022-01908-z (PMC9450370; doi:10.1186/s12934-022-01908-z)
Supplement: Supplementary file 1 — Additional file 1: Table S1. Main plasmids used in the study. Table S2. Primers used in this study. Table S3. Synthesized gene sequence. [file 12934_2022_1908_MOESM1_ESM.docx]

**Supplementary Information**

**Fusing an exonuclease with Cas9 enhances homologous recombination in *Pichia pastoris***

Kun Zhang^1, 2^, Xingpeng Duan^1, 3^, Peng Cai^1,^, Linhui Gao^1, 4^, Xiaoyan Wu^1, 4^, Lun Yao^1, 5^, Yongjin J. Zhou^1, 5, 6^ *

^1^ Division of Biotechnology, Dalian Institute of Chemical Physics, Chinese Academy of Sciences, Dalian 116023, China;

^2^ Henan Engineering Laboratory for Bioconversion Technology of Functional microbes, College of Life Sciences, Henan Normal University, Xinxiang 453007, Henan, China;

^3^ College of Life Sciences, Liaoning Normal University, Dalian 116029, Liaoning, China;

^4^ University of Chinese Academy of Sciences, Beijing 100049, China；

^5^Dalian Key Laboratory of Energy Biotechnology, Dalian Institute of Chemical Physics, Chinese Academy of Sciences, Dalian 116023, China;

^6^ CAS Key Laboratory of Separation Science for Analytical Chemistry, Dalian Institute of Chemical Physics, Chinese Academy of Sciences, Dalian 116023, China.

Corresponding author:

Prof. Yongjin J. Zhou (E-mail: zhouyongjin@dicp.ac.cn)

Supplementary Table S1. Main plasmids used in the study

| Plasmid name | Genotype | Reference |
| --- | --- | --- |
| pPICZ-Cas9-gFAA1 | pPICZ A-*Bleo^R^*-panARS-( T*_AOX1_*-HDV-gRNA/*FAA1*-HH-P*_HTX1_*-*HsCAS9*-T*_DAS1_* ) | [1] |
| pPICZ-Cas9-gFAA2 | pPICZ A-*Bleo^R^*-panARS-( T*_AOX1_*-HDV-gRNA/*FAA2*-HH-P*_HTX1_*-*HsCAS9*-T*_DAS1_* ) | [1] |
| pPICZ-Cas9-gFAA2-POX1-HFD1 | pPICZ A-*Bleo^R^*-panARS-( T*_AOX1_*-HDV-gRNA/*HFD1* -HH-HDV-gRNA/*POX1*-HH-HDV-gRNA/*FAA2*-HH-P*_HTX1_*-*HsCAS9*-T*_DAS1_* ) | [1] |
| pPICZ-Cas9-gFAA1-T7Exo-C | pPICZ A-*Bleo^R^*-panARS-( T*_AOX1_*-HDV-gRNA/*FAA1*-HH-P*_HTX1_*-*HsCAS9*-*T7Exo*-T*_DAS1_* ) | This study |
| pPICZ-Cas9-gFAA1- λRedExo-C | pPICZ A-*Bleo^R^*-panARS-( T*_AOX1_*-HDV-gRNA/*FAA1*-HH-P*_HTX1_*-*HsCAS9*-*λRedExo*-T*_DAS1_* ) | This study |
| pPICZ-Cas9-gFAA1-EcExoIII-C | pPICZ A-*Bleo^R^*-panARS-( T*_AOX1_*-HDV-gRNA/*FAA1*-HH-P*_HTX1_*-*HsCAS9*- *EcExoIII*-T*_DAS1_* ) | This study |
| pPICZ-Cas9-gFAA1- EXO1-C | pPICZ A-*Bleo^R^*-panARS-( T*_AOX1_*-HDV-gRNA/*FAA1*-HH-P*_HTX1_*-*HsCAS9*-*EXO1*-T*_DAS1_* ) | This study |
| pPICZ-Cas9-gFAA1-MRE11-C | pPICZ A-*Bleo^R^*-panARS-( T*_AOX1_*-HDV-gRNA/*FAA1*-HH-P*_HTX1_*-*HsCAS9*-*MRE11*-T*_DAS1_* ) | This study |
| pPICZ-Cas9-gFAA1-T7Exo-N | pPICZ A-*Bleo^R^*-panARS-( T*_AOX1_*-HDV-gRNA/*FAA1*-HH-P*_HTX1_*- *T7Exo*-*HsCAS9*- T*_DAS1_* ) | This study |
| pPICZ-Cas9-gFAA1- λRedExo-N | pPICZ A-*Bleo^R^*-panARS-( T*_AOX1_*-HDV-gRNA/*FAA1*-HH-P*_HTX1_*-*λRedExo*-*HsCAS9* -T*_DAS1_* ) | This study |
| pPICZ-Cas9-gFAA1-EcExoIII-N | pPICZ A-*Bleo^R^*-panARS-( T*_AOX1_*-HDV-gRNA/*FAA1*-HH-P*_HTX1_*-*EcExoIII*-*HsCAS9* -T*_DAS1_* ) | This study |
| pPICZ-Cas9-gFAA1-EXO1-N | pPICZ A-*Bleo^R^*-panARS-( T*_AOX1_*-HDV-gRNA/*FAA1*-HH-P*_HTX1_*-*EXO1*-*HsCAS9*-T*_DAS1_* ) | This study |
| pPICZ-Cas9-gFAA1-MRE11-N | pPICZ A-*Bleo^R^*-panARS-( T*_AOX1_*-HDV-gRNA/*FAA1*-HH-P*_HTX1_*-*MRE11*-*HsCAS9*-T*_DAS1_* ) | This study |
| pPICZ-Cas9-gFAA2-MRE11-C | pPICZ A-*Bleo^R^*-panARS-( T*_AOX1_*-HDV-gRNA/*FAA2*-HH-P*_HTX1_*-*HsCAS9*-*MRE11*-T*_DAS1_* ) | This study |
| pPICZ-Cas9-gFAA2-HFD1 | pPICZ A-*Bleo^R^*-panARS-( T*_AOX1_*-HDV-gRNA/*HFD1*-HH-HDV-gRNA/*FAA2*-HH-P*_HTX1_*-*HsCAS9*-T*_DAS1_* ) | This study |
| pPICZ-Cas9-gFAA2-HFD1- Mre11-C | pPICZ A-*Bleo^R^*-panARS-( T*_AOX1_*-HDV-gRNA/*HFD1*-HH-HDV-gRNA/*FAA2*-HH-P*_HTX1_*-*HsCAS9*-*MRE11*-T*_DAS1_* ) | This study |
| pPICZ-Cas9-gFAA2-POX1-HFD1-Mre11-C | pPICZ A-*Bleo^R^*-panARS-( T*_AOX1_*-HDV-gRNA/*HFD1*-HH-HDV-gRNA/*POX1*-HH-HDV-gRNA/*FAA2*-HH-P*_HTX1_*-*HsCAS9*-*MRE11*-T*_DAS1_* ) | This study |
| pPICZ-Cas9-gSRS2 | pPICZ A-*Bleo^R^*-panARS-( T*_AOX1_*-HDV-gRNA/*SRS2*-HH-P*_HTX1_*-*HsCAS9*-T*_DAS1_* ) | This study |

Supplementary Table S2. Primers used in this study

| Name | Sequence (5' to 3') |
| --- | --- |
| Backbone-DF | GTTTCGTGTTGTAGTTTTAATATAGTTTGAG |
| Backbone-DR | CCAAAGAAGAAAAGAAAAGTTTAAACG |
| Cas9-DF1 | GTCAGCCCTGCTGTCTCCACCGAG |
| Cas9-DR1 | CATACTCAAACTATATTAAAACTACAACACGAAACGATGGACAAGAAGTACTCCATTGG |
| EcExo3-DF | GACTTCCCGTTTAAACTTTTCTTTTCTTCTTTGGTCTTCTGAAAGTGGCCCAAACTGG |
| EcExo3-DR | CCTCTCTCAGCTCGGTGGAGACAGCAGGGCTGACGGTGGTGGTGGTTCTGGTGGTGGTGGTTCTGGTGGTGGTGGTTCTATGAAATTCGTTTCTTTCAATATCAACGG |
| KpExo1-DF | CCTCTCTCAGCTCGGTGGAGACAGCAGGGCTGACGGTGGTGGTGGTTCTGGTGGTGGTGGTTCTGGTGGTGGTGGTTCTATGGGTGTAACCGGTCTTCTACC |
| KPExo1-DR | CTTCCCGTTTAAACTTTTCTTTTCTTCTTTGGTCCTCTGAATATGAACCTGTCCAGGG |
| KpMre11-DF | CCTCTCTCAGCTCGGTGGAGACAGCAGGGCTGACGGTGGTGGTGGTTCTGGTGGTGGTGGTTCTGGTGGTGGTGGTTCTATGCCACACGTTGACAGAATACTC |
| KPMre11-DR | CTTCCCGTTTAAACTTTTCTTTTCTTCTTTGGACGTCTACTTAGGTTGCCAATCAAAG |
| T7Exo-F | GTTCTGGTGGTGGTGGTTCTATGGCTTTGTTGGACTTGAAGCAATTC |
| T7Exo-R | ACTTTTCTTTTCTTCTTTGGAGGTCTCCACAAATAAATCTCTTTATCAATGAAATTATACTC |
| RedExo-F | GTTCTGGTGGTGGTGGTTCTATGACTCCAGATATCATCCTTCAGAGG |
| RedExo-R | ACTTTTCTTTTCTTCTTTGGTCTCCACTGCTCTCCGAAGACG |
| Test-DF | GTTTACTCTGACCAACTTGGGCG |
| Test-DR | CAGATATTATCATCGCGGCTTACGT |
| SV40-DF | GACTTCCCGTTTAAACTTTTCTTTTCTTCTTTGG |
| Ca9-UF | GGTGGTGGTGGTTCTGGTGGTGGTGGTTCTGGTGGTGGTGGTTCTATGGACAAGAAGTACTCCATTGGGC |
| Mre11-UR2 | GGCAACCTAAGTAGACGTGGTGGTGGTGGTTCTGGTGGTGGTGGTTCTGGTGGTGGTGGTTCTATGGACAAGAAGTACTCCATTGGGC |
| Exo1-UR2 | CAGGTTCATATTCAGAGGAGGTGGTGGTGGTTCTGGTGGTGGTGGTTCTGGTGGTGGTGGTTCTATGGACAAGAAGTACTCCATTGGGC |
| KpExo1-UF | CATACTCAAACTATATTAAAACTACAACACGAAACGATGGGTGTAACCGGTCTTCTACC |
| KPExo1-UR | CCACCAGAACCACCACCACCTCCTCTGAATATGAACCTGTCCAGGG |
| KPMre11-UF | CATACTCAAACTATATTAAAACTACAACACGAAACGATGCCACACGTTGACAGAATACTC |
| KPMre11-UR | CCACCAGAACCACCACCACCACGTCTACTTAGGTTGCCAATCAAAG |
| T7Exo-UF | ACTATATTAAAACTACAACAACTAGTCGAAACGATGGCTTTGTTGGACTTGAAGCAATTC |
| T7Exo-UR | CCACCAGAACCACCACCACCAGGTCTCCACAAATAAATCTCTTTATCAATGAAATTATACTC |
| RedExo-UF | ACTATATTAAAACTACAACAACTAGTCGAAACGATGACTCCAGATATCATCCTTCAGAGG |
| RedExo-UR | CCACCAGAACCACCACCACCTCTCCACTGCTCTCCGAAGACG |
| Exo3-UF | ACTATATTAAAACTACAACAACTAGTCGAAACGATGAAATTCGTTTCTTTCAATATCAACGGACTT |
| Exo1-UF | ACTATATTAAAACTACAACAACTAGTCGAAACGATGGGTGTAACCGGTCTTCTACC |
| Exo3-UR | CCACCAGAACCACCACCACCTCTTCTGAAAGTGGCCCAAACTGG |
| Backbone-UR | TGTTGTAGTTTTAATATAGTTTGAGTATGAGATGGAACTCAG |
| Backbone-UF | GGCTGACCCAAAGAAGAAAAGAAAAGT |
| Test-UF | CCATTCAGTGGAATGTGAACCCAC |
| Test-UR | CTTTTGAGCCGCGTGGCTTC |
| FAA1UP-TF | CATGGCCCAACTGGTTACGAAGA |
| FAA1DN-TR | AGACCGTTTACAGCTAGACAAATTTCAACA |
| FAA2UP-TF | AACCAACAACCATCCGAAGAGGAG |
| FAA2DN-TR | GAGTGTAGGTGATAGCTCGGGTC |
| FAA1UP-R | CAATCGGCTGCTCGCTTTCTTGAAGTTTTCTTTGTACGCGTGTTTG |
| FAA1DN-F | TTCCTTGTTGCCTTCCTGAGGGAGACTAAGATGTAGTTTTTAGACTTGA |
| FAA2UP-F | ACACCCAGTTTGAATAATAATAAGCAGAGCTAC |
| FAA2UP-R | CTTAGTCTCCCTCAGGAAGGCAACAAGGAAACGAGAATGGTA |
| FAA2DN-F | TTCCTTGTTGCCTTCCTGAGGGAGACTAAGATGTAGTTTTTAGACTTGA |
| FAA2DN-R | CATCCTCTCTGATGCAAGTCTCTTTG |
| npgA-seq1-F | AGGATTGAAGTTGCTGCTCTGG |
| MmCAR-seq1-R | TCCTACAGATAGATTCAGAGCCGC |
| MmCAR-seq2-F | TGTGCCAAAGCAGGTCTATCAGC |
| ADH5-seq2-R | TGCAAGTCACTATGACAAACACCAG |
| FAA1UP-F | AGGTATCAAAAGCTGATTTTTATTTGCTATTGTTGATTTAC |
| MmCAR-AOX1t-R | GTTGTAATCAAGAGGATGTCAGAATGCC |
| ngpA-DAS1t-F | ACGGGAAGTCTTTACAGTTTTAGTTAGG |
| TPIp-TEF1p-R | GAATAACTGTCGCCTCTTTTATCTGCC |
| MmCAR-TEF1p-F | GACATGTTGGCGAATAACTAAAATGTATGTAG |
| FAA1DN-R | GCATGGTTATGGTGCCGGTTTGG |
| pHTX-R | TTTGATTTGTTTAGGTAACTTGAACTGGATGTATTAGTTTG |
| pHTX-F | GTTTTAGAGCTAGAAATAGCAAGTTAAAATAAGGCTAG |
| HDV-R | CATATATGCAGATGGAAGTGGTCCCATTC |
| gHFD1-F1 | CAAACGGAACTTCTACAGGTCTTTCGC |
| gHFD1-F2 | GAATGGGACCACTTCCATCTGCATATATGCTTTCGCTGATGAGTCCGTGAGG |
| SRS2-UP-F | GTTGACCTGGAACCGACGTTC |
| SRS2-UP-R | GCAGGCTAATCTGGTGTTTGAATAATTTAGTCTCCTTCCTAAAGTGGTTGACAATGC |
| SRS2-DN-F | ACTAAATTATTCAAACACCAGATTAGCCTGC |
| SRS2-DN-R | GCTGATGGTCTTCTTAGACGAAGAAGTAC |
| SRS2-UP-TF | CGTTCTGTGTACAATTCCCATGTTG |
| SRS2-DN-TR | CCCATTCCTGAAGTGTCGCC |
| SRS2-sgRNA-F | TCCAAACTGATGAGTCCGTGAGGACGAAACGAGTAAGCTCGTCTTTGGAGGGAATAACACCGTGTTTTAGAGCTAGAAATAGCAAGTTAAAATAAGGCTAG |
| SRS2-sgRNA-R | GTTTCGTCCTCACGGACTCATCAGTTTGGATTTGATTTGTTTAGGTAACTTGAACTGGATGTATTAGTTTG |

Supplementary Table S3. Synthesized gene sequence

| Name | Sequence |
| --- | --- |
| *T7Exo* | ATGGCTTTGTTGGACTTGAAGCAATTCTACGAGTTGAGGGAGGGATGCGACGACAAGGGTATCTTGGTCATGGACGGTGACTGGCTTGTCTTCCAAGCTATGAGTGCCGCTGAGTTTGATGCCTCTTGGGAGGAGGAGATCTGGCACAGATGTTGTGACCACGCTAAGGCTAGGCAGATCCTTGAGGACTCTATCAAGTCTTACGAGACTAGAAAGAAGGCTTGGGCTGGAGCTCCAATCGTCCTTGCCTTTACCGACTCTGTCAACTGGAGGAAGGAGCTTGTCGACCCAAACTACAAGGCCAATAGGAAGGCCGTCAAGAAGCCAGTCGGTTACTTCGAGTTCCTTGACGCCTTGTTCGAAAGGGAGGAGTTCTACTGCATTAGAGAACCTATGCTTGAGGGTGATGACGTCATGGGAGTCATCGCCAGTAACCCATCTGCTTTTGGAGCTAGAAAAGCCGTCATCATCAGTTGCGATAAGGACTTTAAAACTATTCCTAACTGCGATTTCCTTTGGTGCACCACCGGTAACATCCTTACCCAGACCGAAGAGTCTGCTGACTGGTGGCACCTTTTCCAGACCATCAAGGGAGACATTACCGACGGATACTCTGGAATCGCTGGATGGGGAGACACCGCCGAGGACTTCCTTAACAACCCATTTATCACCGAGCCTAAGACCTCTGTCTTGAAGTCTGGTAAGAACAAGGGTCAAGAAGTCACCAAGTGGGTCAAAAGAGACCCAGAACCACACGAGACCCTTTGGGACTGCATCAAGTCTATCGGAGCCAAGGCCGGTATGACTGAGGAGGATATCATCAAGCAAGGACAGATGGCTAGAATCTTGAGGTTTAACGAGTATAATTTCATTGATAAAGAGATTTATTTGTGGAGACCT |
| *λRedExo* | ATGACTCCAGATATCATCCTTCAGAGGACCGGAATCGATGTCAGAGCTGTCGAGCAAGGAGATGACGCTTGGCACAAGCTTAGGCTTGGTGTCATCACTGCCAGTGAAGTCCACAACGTCATCGCCAAGCCAAGGTCTGGAAAGAAGTGGCCAGATATGAAGATGTCTTACTTCCACACCCTTTTGGCCGAGGTCTGTACTGGTGTTGCTCCAGAAGTCAACGCCAAGGCTCTTGCTTGGGGAAAGCAGTACGAGAACGACGCCAGAACCCTTTTCGAGTTCACCTCTGGAGTCAACGTCACCGAGTCTCCAATCATTTATAGAGACGAGTCTATGAGAACCGCTTGTAGTCCAGACGGTTTGTGCAGTGACGGAAACGGATTGGAGCTTAAGTGCCCTTTCACCTCTAGGGACTTCATGAAGTTTAGACTTGGTGGATTCGAGGCTATCAAGTCTGCCTACATGGCCCAAGTTCAGTACTCTATGTGGGTCACTAGAAAGAACGCTTGGTACTTCGCCAACTACGACCCTAGGATGAAGAGGGAGGGTCTTCACTACGTCGTTATCGAGAGGGACGAGAAGTACATGGCCTCTTTCGACGAGATCGTCCCAGAATTTATCGAGAAAATGGACGAGGCCCTTGCCGAAATCGGATTCGTCTTCGGAGAGCAGTGGAGA |
| *EcExoIII* | ATGAAATTCGTTTCTTTCAATATCAACGGACTTAGGGCTAGACCTCACCAATTGGAAGCCATCGTCGAGAAGCATCAGCCAGATGTCATCGGTTTGCAAGAGACCAAGGTTCACGACGACATGTTCCCATTGGAGGAGGTCGCCAAGCTTGGTTACAACGTCTTCTACCACGGTCAAAAGGGTCACTACGGAGTCGCTTTGCTTACCAAGGAGACCCCAATCGCCGTTAGAAGGGGATTCCCCGGAGACGACGAGGAAGCTCAGAGAAGGATCATCATGGCCGAGATCCCTAGTTTGTTGGGTAACGTCACCGTCATCAACGGTTACTTCCCTCAAGGAGAGAGTAGGGACCACCCTATCAAGTTCCCAGCTAAGGCCCAGTTCTACCAGAACTTGCAAAATTATCTTGAAACCGAGTTGAAGAGAGATAATCCAGTTCTTATCATGGGAGACATGAATATCTCTCCAACCGACTTGGACATCGGAATCGGTGAGGAGAATAGAAAGAGATGGTTGAGGACCGGTAAGTGCTCTTTCCTTCCAGAAGAGAGGGAGTGGATGGATAGACTTATGTCTTGGGGATTGGTCGACACCTTCAGACATGCCAACCCACAGACCGCCGATAGATTCAGTTGGTTCGATTATAGATCTAAGGGTTTCGACGACAATAGAGGACTTAGGATCGACTTGTTGCTTGCCTCTCAGCCTCTTGCTGAATGCTGCGTCGAAACCGGTATCGACTACGAGATTAGAAGTATGGAGAAGCCATCTGACCATGCTCCAGTTTGGGCCACTTTCAGAAGA |

**References**

1. Cai P, Duan X, Wu X, Gao L, Ye M, Zhou YJ: **Recombination machinery engineering facilitates metabolic engineering of the industrial yeast *Pichia pastoris*.** *Nucleic Acids Res* 2021, **49:**7791-7805.
